# The Last Chance Saloon

**DOI:** 10.3389/fcell.2021.671297

**Published:** 2021-05-14

**Authors:** Ye Hong, Hongtao Zhang, Anton Gartner

**Affiliations:** ^1^Shandong Provincial Key Laboratory of Animal Cell and Developmental Biology, School of Life Sciences, Shandong University, Qingdao, China; ^2^Center for Genomic Integrity, Institute for Basic Science, Ulsan, South Korea

**Keywords:** chromatin bridge, abscission checkpoint, NoCut pathway, TREX1, LEM-3 endonuclease, ANKLE1, chromothripsis, micronuclei

## Abstract

Accurate chromosome segregation requires the removal of all chromatin bridges, which link chromosomes before cell division. When chromatin bridges fail to be removed, cell cycle progression may halt, or cytokinesis failure and ensuing polyploidization may occur. Conversely, the inappropriate severing of chromatin bridges leads to chromosome fragmentation, excessive genome instability at breakpoints, micronucleus formation, and chromothripsis. In this mini-review, we first describe the origins of chromatin bridges, the toxic processing of chromatin bridges by mechanical force, and the TREX1 exonuclease. We then focus on the abscission checkpoint (NoCut) which can confer a transient delay in cytokinesis progression to facilitate bridge resolution. Finally, we describe a recently identified mechanism uncovered in *C. elegans* where the conserved midbody associated endonuclease LEM-3/ANKLE1 is able to resolve chromatin bridges generated by various perturbations of DNA metabolism at the final stage of cell division. We also discuss how LEM-3 dependent chromatin bridge resolution may be coordinated with abscission checkpoint (NoCut) to achieve an error-free cleavage, therefore acting as a “last chance saloon” to facilitate genome integrity and organismal survival.

## Introduction

“A last chance saloon” refers to a bar that allows for the legal consumption of alcoholic beverages just before crossing into another jurisdiction where alcoholic beverages are not allowed. Somehow, this phrase got adopted in the United Kingdom to mean “A difficult situation in which there is one final chance to put it right.” Cells get into such a situation surprisingly often. During mitosis, individual chromosome segregation errors are sufficient to drive extensive genomic rearrangements, which in turn are linked with inherited disease and cancer (Ly et al., 2019). To ensure faithful genome maintenance during cell division, chromosomes have to be properly segregated into daughter cells, which requires the removal of all physical connections between sister chromatids. In addition to cohesins, which act as proteinaceous glue, all chromosome connections formed by DNA have to be equally resolved. Failing to remove those DNA connections may lead to the severing of chromosomes (see below) or cause tetraploidisation due to cytokinesis failure, a large number of tumors showing evidence for whole genome duplication (Fujiwara et al., 2005; Lens and Medema, 2019; Quinton et al., 2021). These DNA linkages come in two flavors, chromatin bridges and ultrafine bridges, with the latter not stainable by conventional DNA dyes (Liu et al., 2014).

In this mini-review, we discuss recent advances in how chromatin bridges arising from different perturbations of DNA metabolism are processed just before cells divide during cytokinesis, as well as pathological and non-pathological outcomes of chromatin bridge processing. Chromatin bridges resulting from the segregation of dicentric chromosomes can be severed by nucleases and also by the mechanical force generated from transient actin-myosin assemblies (Maciejowski et al., 2015; Umbreit et al., 2020). These mechanisms allow for cell cycle progression, but at the expense of massive chromosome instability associated with breakage of chromatin bridges. In contrast, there are mechanisms that facilitate the ordered resolution of chromatin bridges that result from DNA catenation, persistent recombination intermediates, or from loci that remain to be replicated. To facilitate an error-free chromatin bridge resolution, the conserved Aurora B kinase mediated NoCut pathway can delay abscission (Norden et al., 2006; Steigemann et al., 2009; Amaral et al., 2016). This abscission checkpoint allows for more time to facilitate chromatin bridge resolution. However, how bridges are eventually resolved remained enigmatic. The recent discovery of a new mechanism in *C. elegans*, conferred by a conserved midbody-tethered endonuclease LEM-3/ANKLE1, might be part of such a mechanism. LEM-3 can resolve chromatin bridges just before cell division is completed, and act as a “last chance saloon” to protect against the severing of chromosomes and associated genome instability (Hong et al., 2018a).

## Formation of the Chromatin Bridges

**FIGURE 1:**
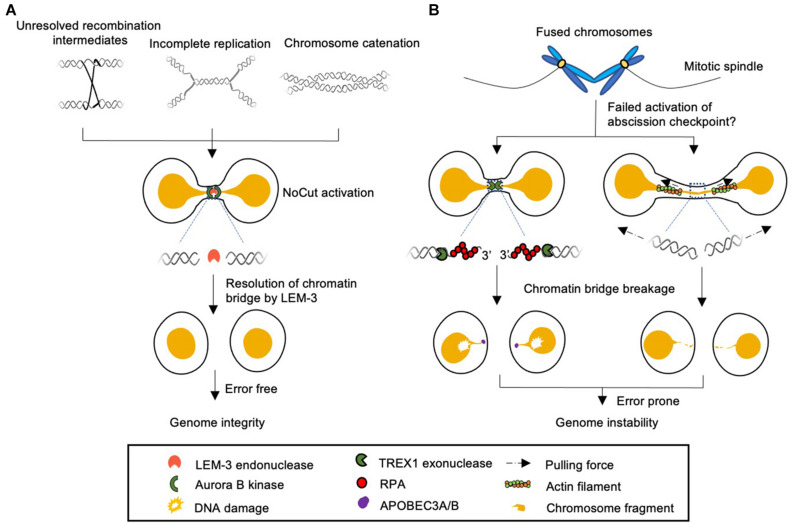
Model of different cellular responses to chromatin bridges during cell division. **(A)** Chromatin bridges arise from incomplete DNA replication, unresolved recombination intermediates, and DNA catenation can be detected by Aurora B kinase dependent NoCut checkpoint. The NoCut checkpoint delays abscission, which allows time for subsequent chromatin bridge resolution. In *C. elegans* chromatin bridges are resolved by the midbody-tethered LEM-3 endonuclease. LEM-3 is likely to be error-free and acts as “a last chance saloon” to protect against the severing of chromosomes and associated genome instability just before cell division is completed. It remains to be elucidated if LEM-3 and its mammalian homolog ANKLE1 are regulated by the NoCut checkpoint pathway. **(B)** Chromatin bridges that fail to trigger the NoCut checkpoint in yeast can be severed by a process that involves the TREX1 exonuclease and APOBEC3A/B deaminases in human cells (left panel). Severing can also occur by actomyosin dependent mechanical force (right panel). These pathways are highly toxic and frequently lead to aberrant chromosomal rearrangements and the formation of micronuclei.

Chromatin bridges were first studied by Barbara McClintock in maize in 1930’s (McClintock, 1941). After X-ray treatment, the broken ends of maize chromosome tend to fuse with one another. When fused chromosomes are pulled toward opposite poles during mitosis, chromatin bridges are created (McClintock, 1941). Alternatively, chromatin bridges can also result from persistent intermediates of recombinational repair such as Holliday junctions, from the intertwining of chromosomes, and from chromosomal loci that have not been replicated by the time cells reach the metaphase-anaphase transition (Finardi et al., 2020; Figure 1A). Regions susceptible to under-replication include centromeres, telomeres and common fragile sites, all containing sequences inherently difficult to replicate (Mankouri et al., 2013). Indeed, incomplete genomic DNA replication is estimated to happen frequently in unperturbed human cells (Moreno et al., 2016).

## Error-Prone Chromatin Bridge Processing

Barbara McClintock noticed that the chromatin bridges can rupture at late anaphase or early telophase, leading to broken chromosome ends (McClintock, 1941). The broken ends of DNA are re-joined to form chromatin bridges in the next cell cycle, which leads to additional rounds of breakage-fusion-bridge (BFB) cycles. Surprisingly, whole genome sequencing of chromosomal end-to-end fusions resulting from telomere deficiency in both *C. elegans* and lymphoblastic leukemia samples revealed surprisingly complex genomic scars (Meier et al., 2014; Maciejowski et al., 2015). In addition to evidence for multiple rounds of BFB cycles, in both cases, initial BFB cycles appear to be followed by an interchromosomal fusion event where chromosomal fragments are randomly inserted into the breakpoint, reminiscent of chromothripsis (Meier et al., 2014; Maciejowski et al., 2015). Chromothripsis is a mutational process by which chromosomal fragments are scattered into a large number of small pieces and reintegrated into one or a few chromosomal loci in random orientation and order (Koltsova et al., 2019).

Recently, live-cell imaging and single cell whole genome sequencing have been combined to investigate a cascade of mutational events initiated by the breakage of chromatin bridges (Umbreit et al., 2020). Live-cell imaging revealed a transient, localized accumulation of actin and myosin on chromatin bridges just before they break. This actin and myosin assembly quickly dissolves after the breakage of chromatin bridges (Umbreit et al., 2020; Figure 1B). Indeed, defects in myosin activation or actin assembly induced by small molecule inhibitors substantially delayed or abrogated chromatin bridge breakage, suggesting that the tension generated by actomyosin force during cell division is required to break chromatin bridges (Umbreit et al., 2020). This mechanism is surprising, given that earlier studies had indicated that mitotic chromosomes are quite flexible and elastic, the pulling force exerted by the spindle on a chromosome being estimated to be one nano-Newton, far too weak to break a chromatin bridge (Houchmandzadeh et al., 1997; Bustamante et al., 2000).

Irrespective, the breakage of chromatin bridges by actomyosin dependent mechanical forces (or experimentally generated by severing with glass capillaries) generates breaks. This is in line with the BFB model, where a reciprocal loss and gain of terminal chromosomal fragments occurs. Some breakpoints show evidence of localized DNA fragmentation. This may lead to a low level of chromothripsis-like rearrangements near the breakpoint (Umbreit et al., 2020). Other breakpoints show evidence of error-prone microhomology mediated replicative DNA repair leading to short tandem repeats (Umbreit et al., 2020). When chromatin bridges enter a second mitosis, excessive damage associated with DNA stubs resulting from chromosome breakage and aberrant DNA replication may trigger an increased preponderance of chromothripsis. In addition, fused chromosomes often missegregate, frequently leading to the formation of micronuclei. Micronuclei can fuse with the nucleus and circular extrachromosomal DNA (ecDNA) contained in micronuclei might serve as a source for DNA fragments, which can be randomly integrated at or close to breakpoints further enhancing chromothripsis (Zhang et al., 2015; Kneissig et al., 2019; Koltsova et al., 2019; Shoshani et al., 2020; Umbreit et al., 2020).

In addition to the direct DNA rupture by mechanical force, the TREX1 exonuclease had also been implicated in severing chromatin bridges. TREX is cytoplasmic and was reported to access chromatin bridges when the nuclear envelope ruptures during interphase (Maciejowski et al., 2015). The exonuclease activity leads to extensive ssDNA formation, which may facilitate DNA breaks as DNA becomes increasingly stretched and fragile (Maciejowski et al., 2015; Figure 1B). In addition, the ssDNA generated by TREX1 can be processed by apolipoprotein B mRNA editing enzyme catalytic polypeptide-like 3B (APOBEC3B) cytidine deaminases, leading to localized C > T and C > G hypermutation close to the breakpoint (Maciejowski et al., 2015, 2020). Such localized hypermutation is referred to as Kataegis and is observed in many cancer types (Chan and Gordenin, 2015). Furthermore, TREX1 expression is correlated with an increased rate and severity of chromothripsis, indicating that the ssDNA generated by TREX1 could also be a substrate for chromothripsis (Maciejowski et al., 2015, 2020). However, depletion of TREX1 does not abrogate the severing of chromatin bridges, consistent with nuclease independent mechanisms allowing for the severing of bridges. Umbreit et al. (2020) failed to observe effects of TREX1 depletion even when using the same cell lines. Irrespective, it remains unclear why nuclease dependent or independent mechanisms have evolved to sever chromatin bridges since this process is associated with massive genome instability. It is tempting to speculate that such mechanisms are restricted to cancer cells which may have lost checkpoints that would trigger the apoptotic demise of affected cells.

## The NoCut Checkpoint Prevents DNA Damage by Delaying Abscission

It has been reported when chromatin bridges are generated by dicentric chromosomes, which are formed by the fusion of two chromosome ends, abscission proceeds normally and finally leads to breakage of chromatin bridges and genomic instability via BFB cycles (McClintock, 1941; Amaral et al., 2016). However, it has been known for more than a decade that the conserved, Aurora B kinase mediated NoCut pathway is able to delay abscission when chromatin is trapped at the cleavage plane during cytokinesis (Amaral et al., 2017; Figure 1A). This pathway is triggered by the presence of chromatin bridges induced by DNA under-replication, chromosome condensation, or decatenation defects (Norden et al., 2006; Steigemann et al., 2009; Amaral et al., 2016). In contrast, when chromatin bridges are generated by dicentric chromosomes in yeast, abscission proceeds without activation of the NoCut checkpoint, suggesting that the cellular responses to different chromatin bridges are distinct (Amaral et al., 2016; Figure 1B).

Cytokinesis typically occurs at the end of mitosis when a contractile ring forms a cleavage furrow and splits one cell into two daughter cells. Chromosome segregation and cytokinesis must therefore be tightly coordinated to ensure that cytokinesis proceeds only after all chromosome are removed from the path of the cleavage furrow. The NoCut pathway delays abscission, allowing for more time to facilitate chromatin bridge resolution. Conversely, the inactivation of abscission checkpoint causes accelerated abscission, which leads to chromatin bridge breakage. The abscission delay is achieved by the persistent activation of the Aurora B kinase at the midbody in cells with chromatin bridges. Aurora B is activated by Cdc-like kinases (CLKs) at the midbody (Petsalaki and Zachos, 2016). Activated Aurora B phosphorylates the “endosomal sorting complex required for transport III” (ESCRT III) subunit CHMP4C and the “mitotic kinesin-like protein 1” (MKLP1) (Steigemann et al., 2009; Carlton et al., 2012). Phosphorylated MKLP1 is thought to stabilize the intercellular canal and block abscission, while phosphorylated CHMP4C can interact with ANCHR (Abscission/NoCut Checkpoint Regulator) to sequester another ESCRT machinery component, the VPS4 ATPase, away from abscission sites, therefore inhibiting abscission and preventing breakage of chromatin bridges during cytokinesis (Steigemann et al., 2009; Thoresen et al., 2014). In contrast, the Aurora B-dependent abscission checkpoint is antagonized by protein phosphatase 1 (PP1) (Bhowmick et al., 2019). Notably, a human polymorphism in CHMP4C, which disrupts the abscission checkpoint, causes an increased level of DNA damage and is associated with increased incidence of multiple cancer types (Sadler et al., 2018). The cancer susceptibility caused by CHMP4C^*T232*^ is likely due to premature abscission. Depletion of the p53 transcription factor in conjunction with CHMP4CT232 leads to synthetic lethality, suggesting that the CHMP4C-dependent abscission checkpoint provides for additional time for cells to protect against chromosome segregation defects and genome instability (Sadler et al., 2018). A recent study showed that the Mre11-Rad50-Nbs1 (MRN) complex can also be recruited to the midbody. The MRN complex activates the DNA damage checkpoint kinases ATM and Chk2 in the presence of chromatin bridges (Petsalaki and Zachos, 2021). Active Chk2 then phosphorylates INCENP, a component of the chromosome passenger complex (CPC), to promote the recruitment of INCENP to the midbody (Petsalaki and Zachos, 2021). INCENP is essential for proper localization of Aurora B (Honda et al., 2003). Once chromatin bridges have been removed from the cleavage plane abscission occurs and cell division is completed.

## The Last Chance Saloon

The key role of NoCut pathway is to delay abscission in response to chromosome segregation defects. However, the cytokinesis delay mediated by NoCut pathway may not be sufficient to prevent DNA damage caused by chromatin bridges. Cells with chromatin bridges form actin patches, as revealed by accumulation of polymerized F-actin at the base of chromatin bridges (Steigemann et al., 2009; Dandoulaki et al., 2018). These actin patches are thought to stabilize the intercellular canal until the chromatin bridges are resolved (Steigemann et al., 2009). The methionine sulfoxide reductase B2 (MsrB2) promotes actin polymerization at the intercellular canal. Depletion of MsrB2 leads to the destabilization of the canal and the ensuing formation of binucleated cells resulting from the failure to process chromatin bridges (Bai et al., 2020). Formation of actin patches also requires Chk1 and Src kinases. Simultaneous depletion of Chk1 or Src and Aurora B leads to reduced actin patches and increased frequency of broken chromatin bridges, suggesting that Chk1 and Src can cooperate with the Aurora B–mediated abscission checkpoint to prevent breakage of chromatin bridges (Dandoulaki et al., 2018).

The requirement for abscission delay to prevent breakage of chromatin bridges raises the question of whether there are active mechanisms of chromatin bridges resolution to avoid the severing of chromosomes, possibly coupled with the Aurora B-dependent NoCut pathway. Careful examination of chromatin bridges in mammalian cells by correlative light and electron microscopy revealed a constriction in chromatin bridges at the midbody region (Shoshani et al., 2020). In addition, the chromatin bridge DNA and Aurora B kinase which resides at midbody showed a mutually exclusive localization after chromatin bridge cleavage, suggesting that the chromatin bridge resolution takes place at the midbody (Shoshani et al., 2020). Indeed, using *C. elegans* genetics and cell biological approaches we recently uncovered a novel mechanism for the chromatin bridge resolution, conferred by a midbody associated endonuclease called LEM-3 (Hong et al., 2018a). The LEM-3 endonuclease was discovered in a genetic screen for DNA repair genes in *C. elegans* (Dittrich et al., 2012). *lem-3* mutants are viable under normal conditions but are hypersensitive to a variety of DNA damaging reagents known to directly or indirectly lead to DNA double-strand breaks (DSBs) (Dittrich et al., 2012). Genetic analysis indicates that LEM-3 is not involved in any of the known DSB repair pathways (Hong et al., 2018a). Unlike TREX1, LEM-3 accumulates at the midbody during the final stages of cell division commencing from anaphase to the very late stage of cytokinesis (Hong et al., 2018a). Such localization can be observed in unperturbed cell cycles, but this localization is enhanced and prolonged when chromatin bridges occur. When DNA replication, recombination or chromosome condensation is partially compromised in wild type embryos, chromatin bridges become visible, but they disappear before cells complete cytokinesis (Hong et al., 2018a). This is not the case in *lem-3* mutants; where chromatin bridges persist, orderly cell cycle progression fails, and tetrapolidization caused by cytokinesis breakdown can be observed (Hong et al., 2018a). LEM-3 is able to process chromatin bridges caused by various perturbations of DNA metabolism, including incomplete DNA replication, unresolved recombination intermediates, and compromised chromosome condensation, indicating that LEM-3 may have a relatively wide substrate specificity (Hong et al., 2018a; Figure 1A). Consistent with this, biochemical analysis of ANKLE1, the LEM-3 ortholog in human cells, revealed that ANKLE1 can cleave a wild range of branched DNA substrate *in vitro*, including flap structures, splayed Y-junctions and helical junctions (Song et al., 2020). The broad substrate specificity, together with the subcellular localization of LEM-3 at the midbody, is in line with its function to process chromatin bridge associated with various perturbations of DNA metabolism at very late stage of cell divisions.

LEM-3 partially colocalized with AIR-2, the Aurora B homolog in *C. elegans*, at the midbody and AIR-2 is required for LEM-3 localization (Hong et al., 2018a). Bioinformatic analysis revealed that LEM-3 contains a conserved AIR-2 phosphorylation motive (K/R; K/R; X0-2; S/T) (Cheeseman et al., 2002; Hong et al., 2018a). Changing the corresponding serines, Ser192 and Ser194 to alanine’s diminished the midbody localization of LEM-3 (Hong et al., 2018a). In addition, using a phospho-specific antibody raised against a peptide containing both phosphorylated S192 and S194, LEM-3 phosphorylation was detected on the midbody in the wild type, but not in the *lem-3 S192A S194A* and *lem-3* mutants, suggesting that the stable localization of LEM-3 at the midbody is likely to be regulated by AIR-2 kinase (Hong et al., 2018a). However, it is unknown how LEM-3 phosphorylated by AIR-2 is recruited to the midbody. Furthermore, it remains unclear if and how resolution of chromatin bridges by LEM-3 is coordinated with the AIR-2 mediated NoCut checkpoint. One possibility is that the nuclease activity of LEM-3 could be regulated by the NoCut checkpoint. Future studies will be required to determine whether the resolution of chromatin bridge by LEM-3 happens only after the NoCut checkpoint dependent inhibition of abscission.

The localization and activity of *C elegans* LEM-3 are highly regulated *in vivo*. Accumulation of LEM-3 at the midbody only occurs at the late stage of mitosis and requires the formation of the central spindle (Hong et al., 2018a). While the localization of endogenous ANKLE1 nuclease still needs to be determined, the spatiotemporal control of nuclease activity is likely to be very important as LEM-3 and ANKLE1 are excluded from the nucleus, and ectopic ANKLE1 nuclear expression leads to chromosome fragmentation (Brachner et al., 2012). Furthermore, LEM-3 specifically acts at the midbody. Ends of resolved chromatin bridges commencing to retract from the midbody (Hong et al., 2018a). The processing of chromatin bridges by LEM-3 has the potential to quickly relieve the tension generated by mitotic force, avoiding chromosome shattering. Interestingly, LEM-3 is able to process chromatin bridges to promote embryonic viability. When DNA replication or chromosome condensation are only weakly perturbed bridges that form in wild type embryos are resolved, while they persist in *lem-3* mutants. Conversely, under those conditions worm development, which involves more than a thousand cell divisions is compromised in *lem-3* mutants, but not in the wild type (Hong et al., 2018a). It remains unclear how the cleavage of chromatin bridges by LEM-3 nuclease promotes embryonic viability. One possibility is that the DSBs generated by LEM-3 are efficiently repaired by DNA repair pathways in the next cell cycle. Indeed, co-depletion of LEM-3 and proteins involved in three major DSB repair pathways; BRC-1 dependent homologous recombination (HR), polymerase Theta (POL-Q) mediated end-joining and LIG-4 dependent non-homologous end-joining, results in synthetic lethality upon IR treatment (Hong et al., 2018a).

## Outlook

It is important to note that the cellular responses to chromatin bridges arise from a variety of perturbations of DNA metabolism are different. For example, while chromatin bridges arise from DNA replication stress, defective chromosome condensation and decatenation are able to trigger the NoCut checkpoint, the NoCut checkpoint fails to be activated when the chromatin bridges were generated by dicentric chromosomes (Amaral et al., 2016). This is consistent with recent findings that chromatin bridges generated by dicentric fusion chromosomes are highly toxic, frequently lead to aberrant chromosomal rearrangements, and the formation of micronuclei (Maciejowski et al., 2015; Umbreit et al., 2020).

In budding yeast, the NoCut checkpoint prevents damage specifically to chromatin bridges generated by DNA replication stress and promotes survival (Amaral et al., 2016). In contrast, the chromatin bridges caused by condensation defects cannot be resolved (Amaral et al., 2016). In *C. elegans*, LEM-3 is able to resolve chromatin bridges that arise from incomplete DNA replication, unresolved recombination intermediates, and compromised chromosome condensation (Hong et al., 2018a). Since LEM-3 is conserved in metazoans only, the underlying molecular mechanism of chromatin bridge resolution could be different. Unlike aberrant chromatin bridge resolution, which leads to non-programmed breaks that cannot be repaired properly, the chromatin bridge cleavage by LEM-3 promotes progeny viability and is likely to be error-free, suggesting that LEM-3 may process chromatin bridges in a sophisticated way to facilitate genome integrity (Hong et al., 2018a). Further studies are needed to elucidate the downstream repair pathways responsible for protecting DNA damage after resolution by LEM-3.

It will be interesting to investigate whether the function of LEM-3 is conserved in mammalian cells. *Ankle1* mutant mice develop normally and no overt DNA repair defects could be observed (Braun et al., 2016). We speculate that a phenotype might be hidden by redundancy. For instance, mice carrying mutations of the *Mus81* and *Slx1* nuclease, which act in conjunction to cut Holliday junctions are viable as is the case of *Gen1* mutants, GEN1 being a canonical Holliday junction resolvase (Castor et al., 2013; Wang et al., 2016). MUS81/SLX1 and GEN1 process persistent recombination intermediates in G2/M and anaphase, respectively (Matos et al., 2011), before LEM-3 appears to act. When *Slx1*/*Mus81* and *Gen1* mutations are combined, embryos die and genome integrity at the cellular level is perturbed (Sarbajna et al., 2014). It is likely that synthetic phenotypes will occur when *Ankle1* mutation is combined with *Slx1* or *Gen1* mutations. Indeed *C*. *elegans slx-1 lem-3* and *mus-81 lem-3* double mutants are lethal, hinting that LEM-3/ANKLE1 is required to process DNA linkages that escaped processing by SLX-1/MUS-81 (Hong et al., 2018b).

A region of human chromosome 13, including *ANKLE1* and a second metabolic gene *ABHD8*, has been shown to be associated with increased risk and severity of breast and ovarian cancer by genome wide association studies (Lawrenson et al., 2016). Recent research using whole exome sequencing of breast cancer samples further indicated that mutation of *ANKLE1* may contribute to the susceptibility to breast cancer (Bakshi et al., 2020). Indeed, the BRCA1 homolog BRC-1 and LEM-3 act synergistically in *C. elegans* to promote genomic stability (Hong et al., 2018a). Therefore, it will be interesting to see whether ANKLE1 expression or function is compromised in those polymorphisms associated with breast and ovarian risk. ANKLE1 has also been indicated as a potential colorectal cancer susceptibility gene (Tian et al., 2020). The expression level of ANKLE1 is lower in colorectal cancer tumors compared with normal tissues (Tian et al., 2020). Knockdown of *ANKLE1* leads to increased cell proliferation and formation of micronucleated cells, suggesting that ANKLE1 could act as a tumor suppressor by maintaining genomic stability (Tian et al., 2020). Future studies are required to determine whether this is related to failure of orderly processing of chromatin bridges by ANKLE1 in cancer cells.

In summary, the midbody associated endonuclease LEM-3 can be regulated by Aurora B kinase and is required for chromatin bridge resolution (Figure 1A). LEM-3 dependent bridge resolution acts as a last chance saloon to facilitate genome integrity and organismal survival. It will be interesting to investigate if regulation of LEM-3/ANKLE1 nuclease is directly linked to the NoCut pathway. Furthermore, future studies on ANKLE1 and its interaction network may have clinical implications in breast, ovarian and colorectal cancers.

## Author Contributions

All authors listed have made a substantial, direct and intellectual contribution to the work, and approved it for publication.

## Conflict of Interest

The authors declare that the research was conducted in the absence of any commercial or financial relationships that could be construed as a potential conflict of interest.
